# Supplementary material for: Assessing flash flood erosion following storm Daniel in Libya
Source: Nat Commun. 2024 Aug 20;15:6493. doi: 10.1038/s41467-024-49699-8 (PMC11336083; doi:10.1038/s41467-024-49699-8)
Supplement: Supplementary file 1 — Supplementary Information [file 41467_2024_49699_MOESM1_ESM.pdf]

Supplementary Information for "Assessing Flash Flood Erosion Following Storm Daniel in Libya"

Jonathan C.L. Normand, Essam Heggy

Comparison of the flood erosion/deposits with the land cover

First, we simplify the Food and Agriculture Organization (FAO) classes of the land cover map [75] for the ROI to facilitate comparison with our flood erosion/deposits map derived from CCD-PCA. The simplified classes are detailed in Supplementary Table 1. Notably, shrublands (37.7 %), grasslands–croplands (10.2 %), and bare areas (50.3 %) account for 98.2 % of the ROI's surface area, while urban areas contribute only to 1.4%. Moreover, Supplementary Table reveals that flood erosion primarily affects shrublands (54.3 %), bare areas (32 %), and croplands (12.3 %); however, we cannot interpret these numbers because of the high spatial correlation between the land cover distribution and the storm trajectory within the ROI, from North-West to South-East, as depicted in Supplementary Fig. 1, where shrublands received most of the precipitation. To mitigate this correlation, we normalize these values for each class to obtain the magnitude of surface change within each land cover class. Thus, 25.8 % of the shrublands, 21.6 % of the croplands, 11.4 % of the bare areas, and 10.8 % of urban areas are affected by flood erosion/deposits/damages. The last figure is significant compared to the 1.4 % urban coverage; this illustrates the catastrophic impact of the landfall on infrastructures and human lives.

FAO class definition [75]:

- 130: Closed to open (>15%) (broadleaved or needleleaved, evergreen or deciduous) shrubland (<5m)
- 200: Bare areas
- 30: Mosaic vegetation (grassland/shrubland/forest) (50-70%) / cropland (20-50%)
- 201: Consolidated bare areas
- 11: Post-flooding or irrigated croplands (or aquatic)
- 150: Sparse (<15%) Woody Vegetation/Herbaceous Sparse Vegetation
- 20: Mosaic cropland (50-70%) / vegetation (grassland/shrubland/forest) (20-50%)
- 13: Irrigated herbaceous crops
- 190: Artificial surfaces and associated areas (Urban areas >50%)
- 14: Rainfed croplands
- 210: Water Bodies
- 134: Closed to open broadleaved deciduous shrubland

- 50: Closed (>40%) broadleaved deciduous forest (>5m)
- 151: Sparse grassland
- Unclassed: CCD pixels that do not fall into a FAO class

Supplementary Table 1. Simplified land cover within the emerged parts of the ROI (ROI km^2 and ROI %), within the erosion areas identified by CCD–PCA (CCD km^2, CCD %, ex: 54.3 % of the eroded areas are located within the Shrublands), and the erosion within each land cover class (CCD ⊆ FAO, ex: 25.8 % of shrublands within the ROI are affected by flood erosion).

	**FAO code**	**ROI km^2**	**ROI %**	**CCD km^2**	**CCD %**	**CCD⊆FAO %**
**Shrubland (50-100%) / Grassland–Cropland (0-50%)**	130 & 134 & 30	1815.25	37.7	467.76	54.3	25.8
**Bare areas / sparse vegetation**	200 & 201 & 150	2419.48	50.3	275.65	32.0	11.4
**Grassland–Cropland (50-100%)/ Shrubland (0-50%)**	11 & 13 & 14 & 20	492.63	10.2	106.29	12.3	21.6
**Artificial surfaces and associated areas (Urban areas >50%)**	190	66.28	1.4	7.14	0.8	10.8
**Water Bodies**	210	15.03	0.3	1.23	0.1	8.2
**Closed (>40%) broadleaved deciduous forest (>5m)**	50	0.24	0.0	0.13	0.0	54.2
**Sparse grassland**	151	0.40	0.0	0.02	0.0	5.0
**Unclassed**	—	—	—	3.27	0.4	—
	**TOTAL**	**4809.31**	**100**	**861.49**	**100**	—

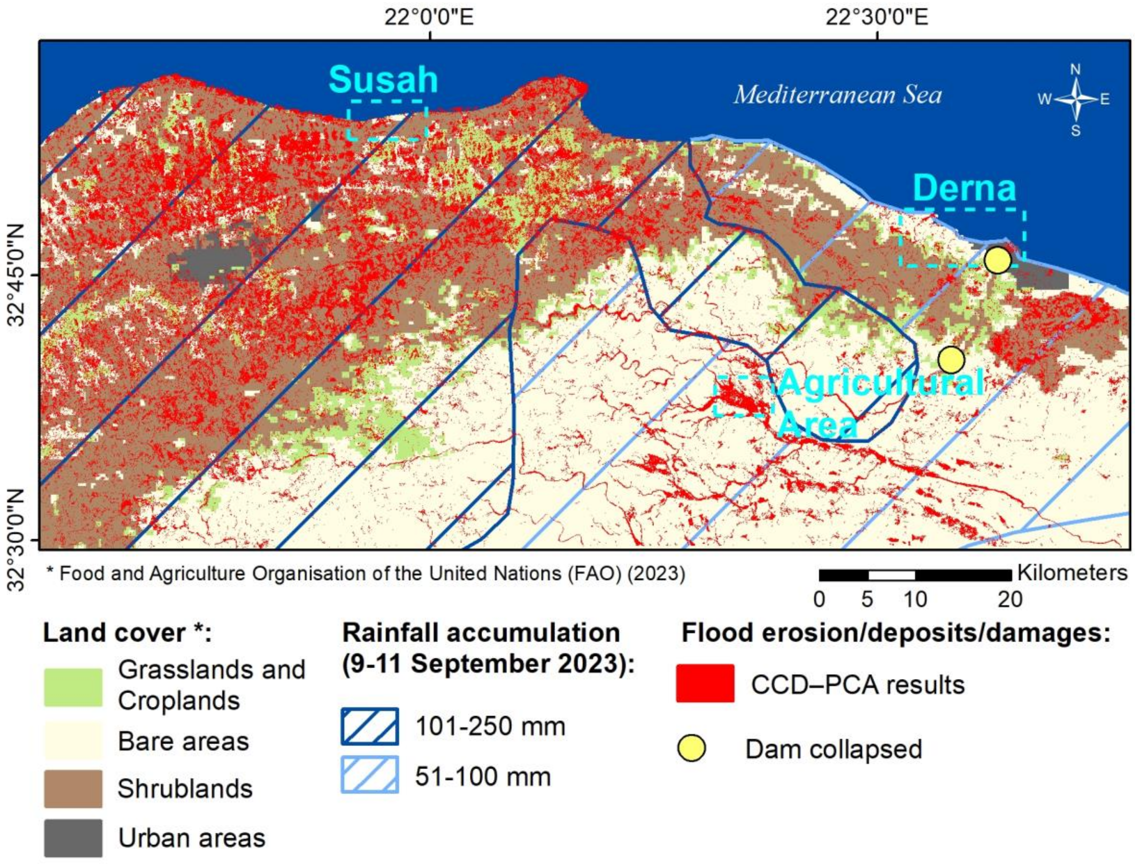

Supplementary Fig. 1. **Simplified land cover [75] and flood erosion/deposits/damages derived from CCD–PCA.** In particular, 54.3 % of the eroded areas (in red) are located within the Shrublands (in brown) and 25.8 % of shrublands were eroded by the flood (see Supplementary Table 1). Moreover, 32 % of the eroded areas are located within the bare areas (in yellow) and 11.4 % of bare areas were eroded.

List of satellite images used for CCD–PCA processing and visualization

Supplementary Table 2 and Supplementary Table 3, presented below, enumerate the SAR and Multispectral images utilized in this study to identify and visualize flood erosion. Moreover, Supplementary Table 4 lists the selection of InSAR pairs used in the CCD method.

Supplementary Table 2. C-band SAR images used in the CCD–PCA.

Date (yyyy–mm–jj)	Satellite name	Direction, Polarization and Level	Relative orbit number	Scene ID	Wavelength
2023–06–21	Sentinel–1A	Desc., VV, L1	7	386783	5.6 cm
2023–07–03	Sentinel–1A	Desc., VV, L1	7	388143	5.6 cm
2023–07–15	Sentinel–1A	Desc., VV, L1	7	389532	5.6 cm
2023-07–27	Sentinel–1A	Desc., VV, L1	7	390906	5.6 cm
2023–08–08	Sentinel–1A	Desc., VV, L1	7	392297	5.6 cm
2023–08–20	Sentinel–1A	Desc., VV, L1	7	393833	5.6 cm
2023–09–25	Sentinel–1A	Desc., VV, L1	7	398421	5.6 cm
2023–10–07	Sentinel–1A	Desc., VV, L1	7	399956	5.6 cm

Supplementary Table 3. C-band SAR and multispectral images used for visualization only.

Date (yyyy–mm–jj)	Satellite name	Type and Level	Relative orbit Number & Scene ID	Wavelength
2023–09–13	Sentinel–1A	SAR, L1	7 & 396898	5.6 cm
2023–07–09	Sentinel–2B	Multispectral, L2A	50 & 34SFB<br>50 & 34SEA<br>50 & 34SFA<br>50 & 34SEB	Band 4 (665 nm),<br>Band 3 (560 nm),<br>Band 2 (490 nm)
2023–09–07	Sentinel–2B	Multispectral, L2A	50 & 34SEB	Band 4 (665 nm),<br>Band 3 (560 nm),<br>Band 2 (490 nm)
2023–09–12	Sentinel–2B	Multispectral, L2A	50 & 34SFB<br>50 & 34SEA<br>50 & 34SFA<br>50 & 34SEB	Band 4 (665 nm),<br>Band 3 (560 nm),<br>Band 2 (490 nm)
2023–09–15	Sentinel–2A	Multispectral, L2A	50 & 34SEB	Band 4 (665 nm),<br>Band 3 (560 nm),<br>Band 2 (490 nm)

Supplementary Table 4. Coherence pairs used in the CCD–PCA analysis.

InSAR coherence pair		CCD–PCA with storm occurrence	CCD–PCA without storm occurrence	Interval (days)	Perpendicular baseline (m)
2023–06–21	2023–08–08	×	×	48	68
2023–07–03	2023–07–27	×	×	24	45
2023–07–03	2023–07–15	×	×	12	50
2023–07–27	2023–08–20	×	×	24	53
2023–07–15	2023–08–20	×	×	36	41
2023–08–20	2023–09–25	×		36	41
2023–07–27	2023–10–07	×		72	23

Validation of CCD–PCA in Derna and Susah

We conduct this quantitative validation analysis to compare our damage map derived from CCD–PCA with the damage assessment maps provided by CEMS, including building, bridge and road damage assessments. To start, we establish a spatial unit of the same size for the maps, opting to downscale the datasets to a 50 m × 50 m spatial unit. Then, we intersect (refer to (4)) the occurrences of radar changes with the occurrences of any damage within the CEMS maps for each downscaled cell. It is worth noting that we conduct this spatial intersection twice: first, by considering destroyed buildings, and second, by considering both damaged and destroyed buildings.

From this spatial comparison, we extract and list in Supplementary Table 5 the “true-detection”, “omission“, and false detection.”

$$\text{CCD Validation} = \text{CCD} \cap (\text{Building} \cup \text{Bridge} \cup \text{Road}) \qquad (4)$$

Where CCD is the damage map derived from CCD–PCA, “building”, “bridge”, and “road” refer to the CEMS maps of damaged/destroyed buildings, destroyed bridges, and destroyed roads, respectively.

The CEMS dataset encompasses the two following damage scenari:

- Case 1: With damaged or destroyed buildings, destroyed bridges, damaged and destroyed roads
- Case 2: With destroyed buildings, destroyed bridges, damaged and destroyed roads

The validation of our CCD–PCA with the CEMS dataset in Derna (see Supplementary Fig. 2) shows that the CCD–PCA is valid for 60.9 % of the area for Case 1 (see Supplementary Fig. 2c) and 65.5 % for Case 2 (see Supplementary Fig. 2d). Moreover, in Susah, we show that the CCD–PCA is valid for 42 % of the area for Case 1 (see Supplementary Fig. 3b) and 64.5

% for Case 2 (see Supplementary Fig. 3d). This validation confirms that “destroyed buildings” are more detectable from the radar CCD–PCA method than “damaged buildings”. We attribute this sensitivity to the difference in phase variability for each class of damage magnitude. Specifically, “destroyed buildings” are associated with significant phase spatial variability, while “damaged buildings” exhibit a more subtle phase spatial variability.

Supplementary Table 5. CCD–PCA validation in Derna within the flooding extent, for two scenari.

			**Surface changes derived from CCD–PCA**				
			True Detection (occurrence)	True Detection (no damage)	Missing detection (Omission)	False detection (Commission)	Total
**CEMS map**	Case 1: With damaged or destroyed buildings, destroyed bridges, damaged and destroyed roads	Bins	634	89	385	79	1187
		%	60.9 %		32.4 %	6.7 %	100 %
	Case 2: With destroyed buildings, destroyed bridges, damaged and destroyed roads	Bins	368	409	65	345	1187
		%	65.5 %		5.5 %	29 %	100 %

Supplementary Table 6. CCD–PCA validation in Susah within the flooding extent, for two scenari.

			**Surface changes derived from CCD–PCA**				
			True Detection (occurrence)	True Detection (no damage)	Missing detection (Omission)	False detection (Commission)	Total
**CEMS map**	Case 1: With damaged or destroyed buildings, destroyed bridges, destroyed roads	Bins	751	349	1044	467	2611
		%	42 %		40 %	18 %	100 %
	Case 2: With destroyed buildings, destroyed bridges, destroyed roads	Bins	326	1356	37	892	2611
		%	64.5 %		1.5 %	34 %	100 %

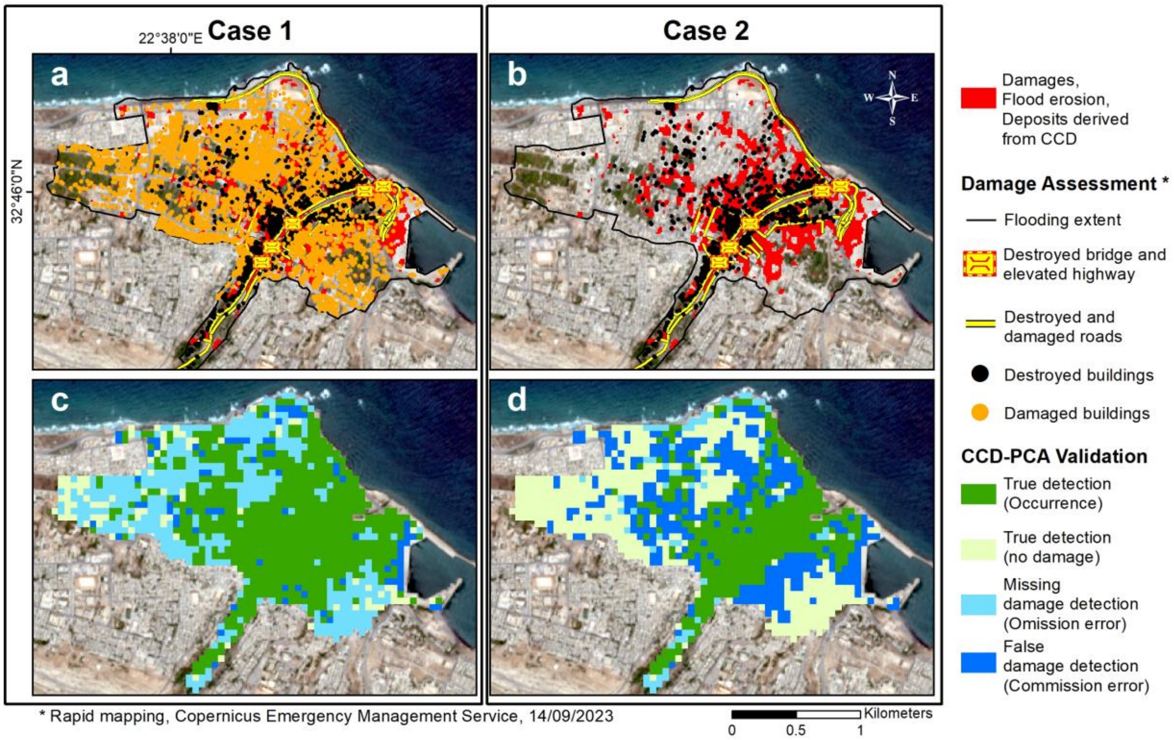

Supplementary Fig. 2. **Validation of CCD–PCA in Derna for two scenari within the flooding extent: Case 1 and Case 2. a.** Damaged and destroyed buildings, destroyed bridges, damaged and destroyed roads (Case 1) assessed by CEMS and overlapped over the CCD–PCA observations. **b.** Damaged buildings only, destroyed bridges, damaged and destroyed roads (Case 2), assessed by CEMS and overlapped over the CCD–PCA observations. **c.** Validation of CCD–PCA for Case 1. **d.** Validation of CCD–PCA for Case 2. The true detection–occurrence corresponds to the validation of the CCD–PCA with the infrastructure damages and/or destructions assessed by CEMS within a 50 m × 50 m cell, while true detection–no damage, signifies the absence of damage for both CEMS and CCD–PCA observations. The CCD–PCA is valid for 60.9 % of the area in Case 1 (frame c), and 65.5 % in Case 2 (frame d).

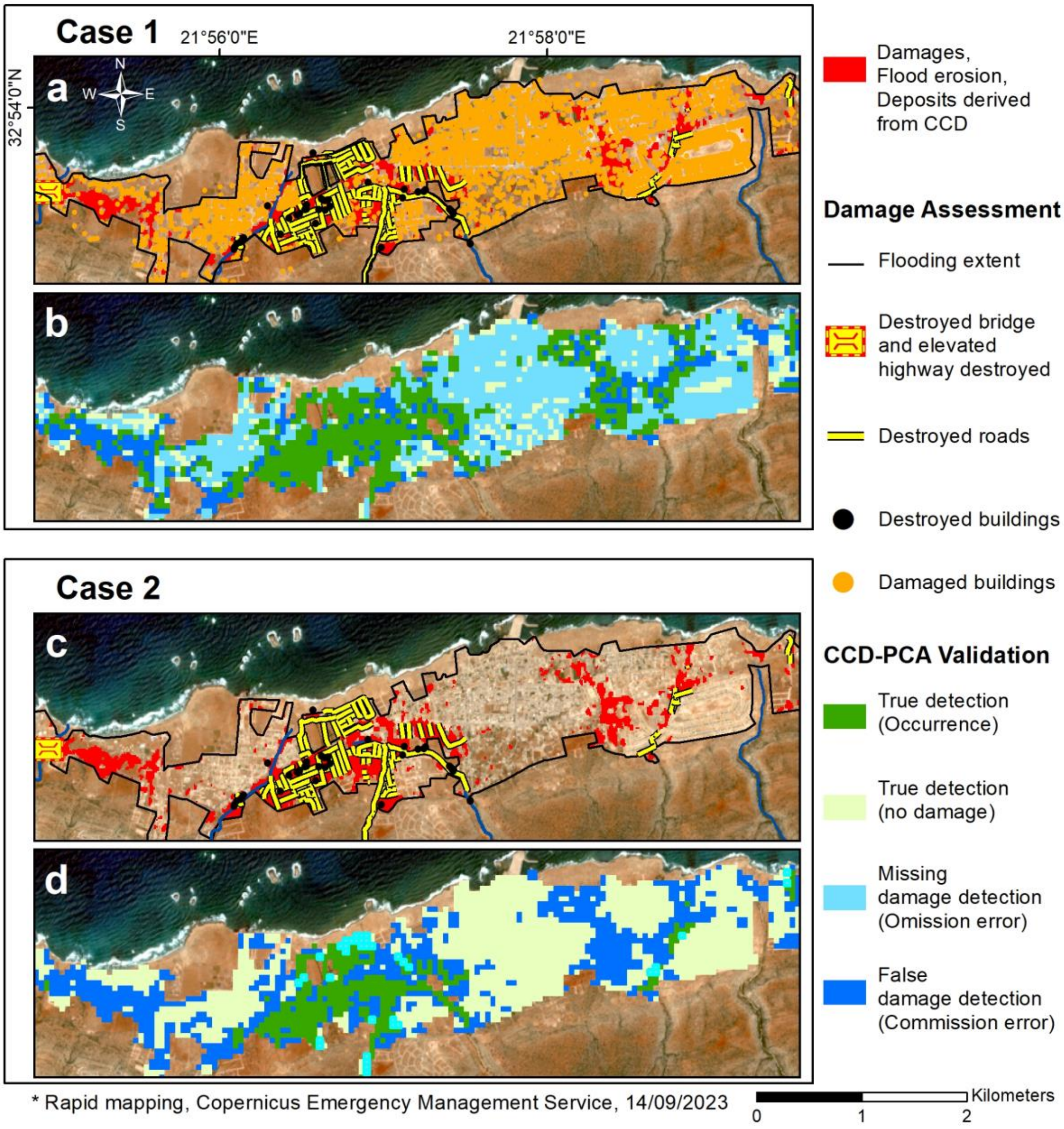

Supplementary Fig. 3. **Validation of CCD–PCA in Susah for two scenari within the flooding extent: Case 1 and Case 2. a.** Damaged and destroyed buildings, destroyed bridges, damaged and destroyed roads (Case 1) assessed by CEMS and overlapped over the CCD–PCA observations. **b.** Damaged buildings only, destroyed bridges, damaged and destroyed roads (Case 2), assessed by CEMS and overlapped over the CCD–PCA observations. **c.** Validation of CCD–PCA for Case 1. **d.** Validation of CCD–PCA for Case 2. The true detection–occurrence corresponds to the validation of the CCD–PCA with the infrastructure damages and/or destructions assessed by CEMS within a 50 m × 50 m cell, while true detection–no damage, signifies the absence of damage for both CEMS and CCD–PCA observations. The CCD–PCA is valid for 42 % of the area in Case 1 (frame b), and 64.5 % in Case 2 (frame d).

Identification of the intrinsic CCD–PCA error

Herein, we define 'pre-storm erosion' as the identification of erosion during a period when no rain occurred; hence, the loss of coherence can be due to vegetation, sediment deposition through wind, weathering (chemical and physical), residual noise from surface roughness and other typical factors that could affect the coherence such as spatial decorrelation.

In our methodology, we already discriminate the 'background coherence noise' by comparing in a PCA a "dry" coherence pair overlapping a rainstorm and containing the erosion information, with a "dry" coherence pair showing no event. However, we also perform a PCA over the summer when no rainstorm occurred to quantify any possible commission error, false-positive identification of erosion that is an intrinsic error of our CCD–PCA method (see coherence pairs in Supplementary Table 4). These commission and false-positive errors should not be confused with those discussed in the "Validation of CCD–PCA" section in Method, which pertains to a comparison with ground-truthing datasets. Here, the commission and false-positive errors address the signal variability, inherent to the CCD–PCA method itself.

Supplementary Fig. 4 shows the identification of erosion (in red) from the first PCA, the false-positive identification of erosion (in blue) from the second PCA, and the common area (in stripes).

$$P(E_{noise}) = P(NE_{coh} \subseteq E_{coh}) = \frac{P(E_{coh} \subseteq ROI) \cdot P(NE_{coh} \subseteq E_{coh})}{P(E_{coh} \subseteq ROI)} = \frac{P(E_{coh} \subseteq ROI \cap NE_{coh} \subseteq E_{coh})}{P(E_{coh} \subseteq ROI)} = \frac{0.023}{0.18} = 0.13 = 13\% \quad (5)$$

Within the emerged lands of the ROI, the flood erosion (E_{coh}) identified represents 18 % of the area, while the pre-storm erosion (NE_{coh}) represents 9.3 %, and the common areas (i.e., false-positive) between the two represent 2.3 %. The probability of false positive (E_{noise}) represents the probability that NE_{coh} falls into E_{coh} noted $P(NE_{coh} \subseteq E_{coh})$. Hence, using (5), we calculate that $P(E_{noise}) = 13\%$.

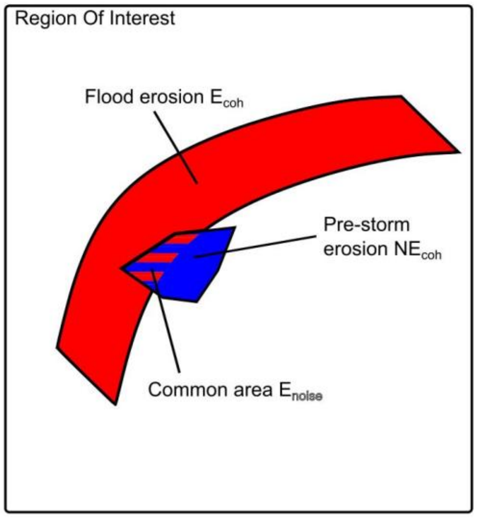

Supplementary Fig. 4. **Assessment of false positive E_{noise}.** The false positive E_{noise} is determined by intersecting flood erosion E_{coh} (in red) with pre-storm erosion NE_{coh} (in blue).

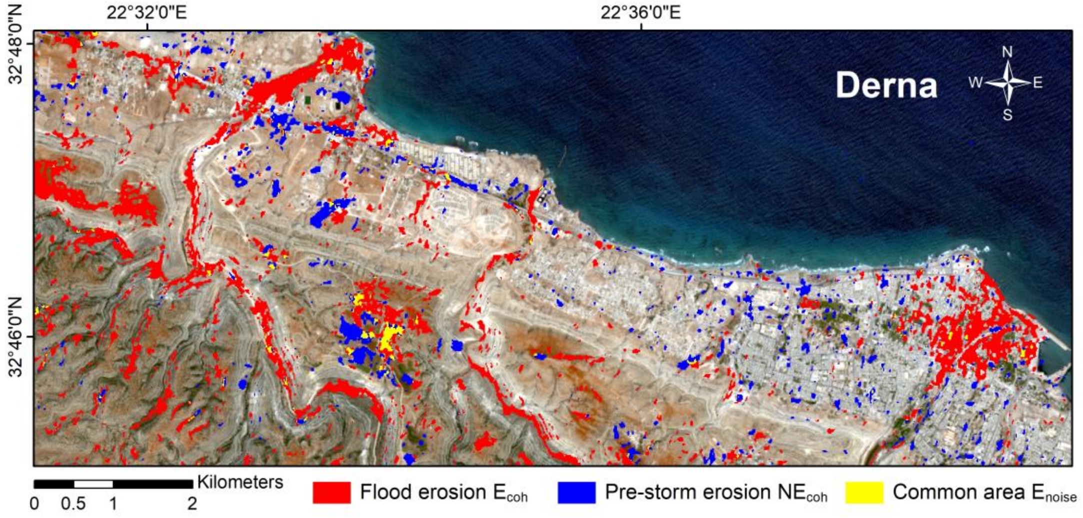

Supplementary Fig. 5. **Identification of false positives E_{noise} flood erosion (commission errors in yellow) in Derna.** The false positive E_{noise} is determined by intersecting flood erosion E_{coh} (in red) with pre-storm erosion NE_{coh} (in blue).

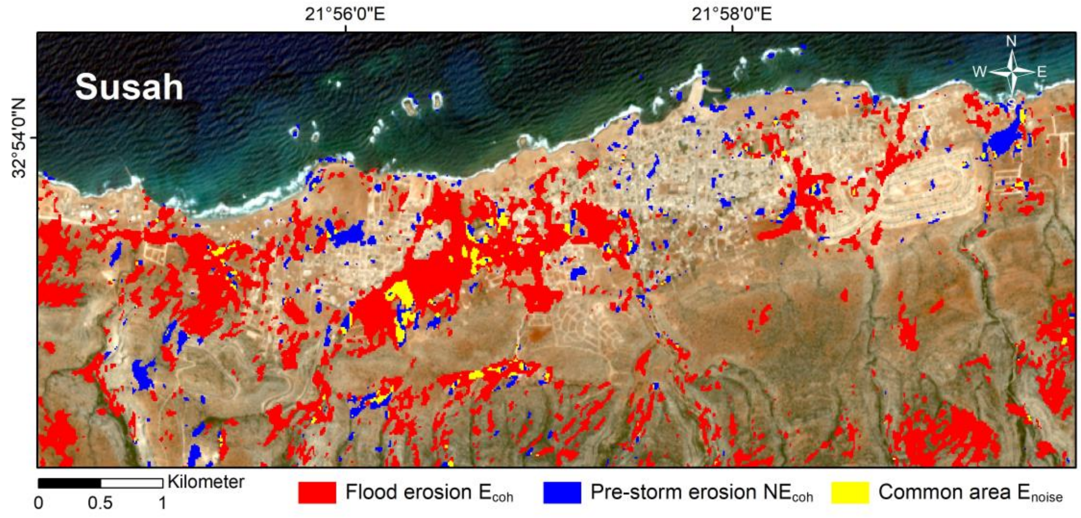

Supplementary Fig. 6. **Identification of false positives E_{noise} flood erosion (commission errors in yellow) in Susah.** The false positive E_{noise} is determined by intersecting flood erosion E_{coh} (in red) with pre-storm erosion NE_{coh} (in blue).

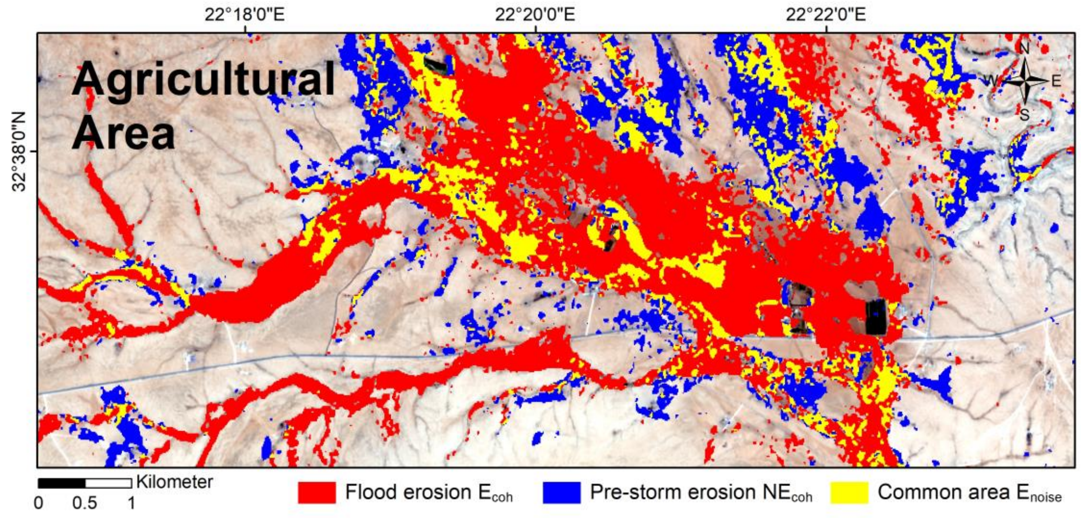

Supplementary Fig. 7. **Identification of false positives E_{noise} flood erosion (commission errors in yellow) in the agricultural area.** The false positive E_{noise} is determined by intersecting flood erosion E_{coh} (in red) with pre-storm erosion NE_{coh} (in blue).

Spatial resolution

After a multilooking of 2 in azimuth direction and 7 in range direction, the Sentinel-1 SLC pixel spacing of 3.9 m × 13.9 m in range and azimuth, respectively, becomes a spatial resolution of 27 m. Then, using the adaptive nonlocal–InSAR (ANL–InSAR) spatial filtering[76] with a kernel varying from 3 to 7, depending on the InSAR variability, we obtain a final spatial resolution ranging from 81 m to 189 m.

Georeferencing and spatial accuracy

Overlapping products derived from radar imagery with optical images presents challenges due to geometric distortions from the layover and shadow effects arising from topography and look angle [77, 78]. While georeferencing with a Digital Elevation Model (DEM) resolves the issue of overlapping, it also introduces distortions by stretching and shrinking radar images in areas of layovers and shadows, which are common in areas characterized by steep terrain, such as canyons, gullies and mountainous regions. This limitation could impact the identification of flood erosion, as it may alter the dimensions of areas experiencing surface changes.

To mitigate the above-mentioned distortion, our first approach is to georeference the radar CCD–PCA products without relying on a DEM but by selecting 118 ground control points (GCPs) distributed over the ROI. This georeferencing results in a root mean square (RMS) error of 177 m (spatial accuracy). While the georeferencing is suitable for some regions of the ROI, it falls short in other parts where the radar product shows significant spatial inconsistencies. Indeed, by adding this spatial accuracy to the spatial resolution, we obtain an error spanning from 258 m to 366 m.

As a result, we choose to georeference the radar products with the DEM, despite the potential distortion effects limiting the interpretation of flood erosion.

Requirement for effective PCA in the CCD

In our investigation, we base our CCD approach on Principal Component Analysis (PCA), which is primarily designed to capture linear relationships within a dataset.

To assess the linearity of the temporal coherence decorrelation, we produce a coherence time-series based on 69 SAR images (i.e., Sentinel-1A Descending, relative orbit number #7, with a 12-day acquisition interval) for the 2021–07–01 to 2023–09–25 period. We observe that the temporal coherence decorrelation follows a linear decay trend within a period of up to 72–84 days (see Supplementary Fig. 8; the coherence time-series is extracted from our sub-region of interest presented in Supplementary Fig. 9). Beyond this period, the trend transitions to an exponential decay associated with an annual periodic component.

Therefore, applying PCA on coherence pairs with a temporal baseline up to 72 days is acceptable for our sub-region of interest; the first principal component summarizes this linearity in temporal decorrelation, while the second principal component summarizes the remaining residuals (i.e., the CCD itself).

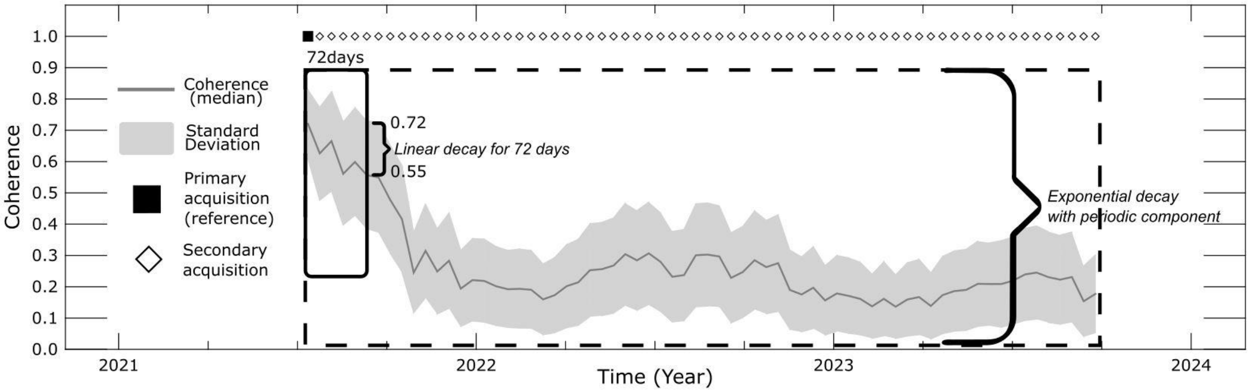

Supplementary Fig. 8. **Coherence Time-Series.** A two-year coherence time-series derived from 69 SAR scenes presents an exponential decay with an annual periodic component. In contrast, short-term coherence time-series spanning up to 72 days presents linear decay, allowing the use of PCA within this time frame. The time-series represents the sub-region of interest mapped in Supplementary Fig. 9.

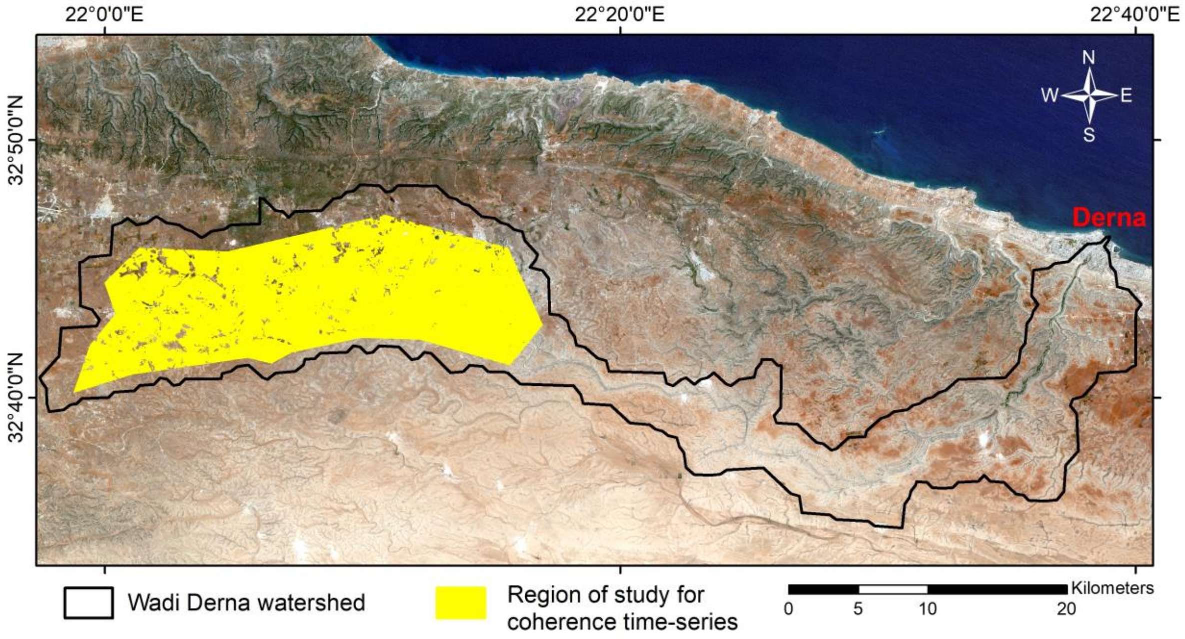

Supplementary Fig. 9. **Region selected to perform a coherence time-series.** The region is located upstream of the Wadi Derna watershed. In Supplementary Fig. 8, we present the coherence time-series generated from this specific region. To ensure data quality within this region, we apply a 0.25 coherence threshold to mask areas with vegetation and other low-coherence areas. This selection is based on coherence measurements from a 12-day pair in July 2023.

Sediment loading assessment

From the criteria outlined in (6) and the threshold values provided in Supplementary Table 7, we delineate the erosion hotspots over the ROI across a grid of 2 km × 2 km bin size (see Supplementary Fig. 10).

$$\mathrm{Erosion}_{\mathrm{HotSpot}} = \mathrm{H}_{\mathrm{rain}} \times \mathrm{H}_{\mathrm{eros}} \times \mathrm{V}_{\mathrm{slop}} \quad (6)$$

Where H_{rain}, H_{eros}, V_{slop} represent the class values for the 3-day rainfall accumulation hazard, flood erosion hazard, and slope gradient vulnerabilities, respectively.

Finally, the estimation of sediment loading in wadis is achieved by intersecting the areas of erosion hotspots with the wadis locations, as outlined in Supplementary Fig. 11.

$$\mathrm{Sediment\ Loading} = \mathrm{Wadis} \cap \mathrm{Erosion}_{\mathrm{HotSpot}} \quad (7)$$

Supplementary Table 7. Hazard and vulnerability variables characterizing the erosion hotspots. The assessment incorporates three variables within a bin of 2 km × 2 km: rainfall accumulation and observed flood surface changes, representing hazard variables, and topographic slope, representing vulnerability variables. The erosion hotspot areas are determined by multiplying the three variables, as expressed in (6).

	Hazard		Vulnerability
Class	Rainfall accumulation (3 days)	Flood surface change derived from CCD–PCA	Average slope angle
0	< 50 mm	< 5% of the bin	0–3 °
1	> 50 mm	> 5% of the bin	> 3 °

Supplementary Table 8. Determining damage categories in Derna and Susah from CCD–PCA results and CEMS map.

<table>
<tr><td colspan="2" rowspan="2"></td><td colspan="4">Surface changes derived from CCD–PCA</td></tr>
<tr><td>True Detection (occurrence)</td><td>True Detection (no damage)</td><td>Missing detection (Omission)</td><td>False detection (Commission)</td></tr>
<tr><td rowspan="2">CEMS map</td><td>Case 1: With damaged or destroyed buildings, destroyed bridges, damaged and destroyed roads</td><td>High damages</td><td>No damage</td><td>Low damages</td><td>Moderate damages</td></tr>
<tr><td>Case 2: With destroyed buildings, destroyed bridges, damaged and destroyed roads</td><td>High damages</td><td>No damage</td><td>High damages</td><td>Moderate damages</td></tr>
</table>

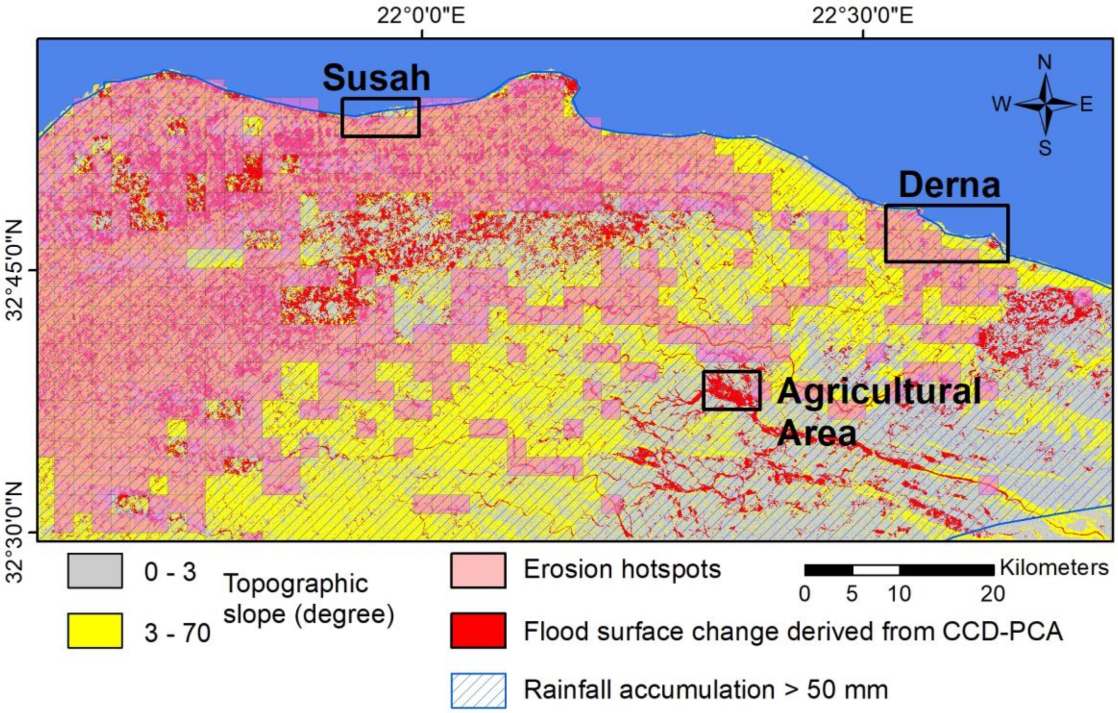

Supplementary Fig. 10. **Erosion hotspots assessment.** Erosion hotspots assessment (in pink) performed within a 2 km × 2 km cell, encompassing hazard (flood surface change derived from CCD in red and rainfall accumulation in dash blue) and vulnerability variables (topographic slopes in yellow).

Identification of wadis

To delineate the wadi networks within the ROI, we employ the following methodology using ArcGIS software. First, we fill any possible topographic depressions in the NASA DEM, a crucial step ensuring continuous run-off flow. Then, using the D8 method, we determine the flow direction from each cell by identifying the steepest gradient among the eight adjacent cells [79]. Next, we compute the flow accumulation for each cell. Finally, we determine the hierarchical order of wadis in the network using the Strahler stream order method [80].

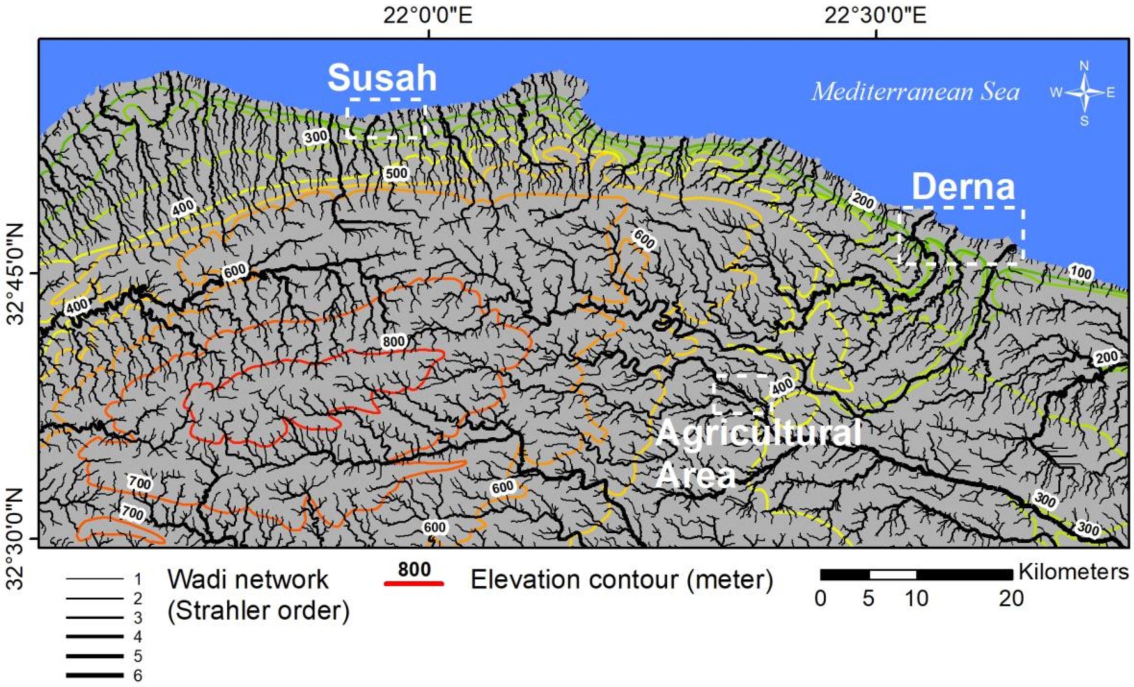

Supplementary Fig. 11. **Wadi network derived from NASA DEM.** The thickness of the lines representing the wadis varies according to their Strahler order. Elevation ranges from 100 m (in green) to 800 m (in red).

References

75 Food and Agriculture Organisation of the United Nations (FAO). Land cover of Libyan Arab Jamahiriya - Globcover Regional (46 classes) (2023).

76 Deledalle, C.-A., Denis, L. & Tupin, F. NL-InSAR: Nonlocal interferogram estimation. IEEE Transactions on Geoscience and Remote Sensing 49, 1441-1452, doi:https://doi.org/10.1109/TGRS.2010.2076376 (2010).

77 Dowman, I. The geometry of SAR images for geocoding and stereo applications. International Journal of Remote Sensing 13, 1609-1617, doi:https://doi.org/10.1080/01431169208904215 (1992).

78 Woodhouse, I. H. Introduction to Microwave Remote Sensing. (CRC Press, 2005).

79 Tarboton, D. G. A new method for the determination of flow directions and upslope areas in grid digital elevation models. Water resources research 33, 309-319, doi:https://doi.org/10.1029/96WR03137 (1997).

80 Strahler, A. N. Quantitative analysis of watershed geomorphology. Eos, Transactions American Geophysical Union 38, 913-920, doi:https://doi.org/10.1029/TR038i006p00913 (1957).
